# Frequency, risk factors, and outcomes of hospital readmissions of COVID-19 patients

**DOI:** 10.1038/s41598-021-93076-0

**Published:** 2021-07-02

**Authors:** Antonio Ramos-Martínez, Lina Marcela Parra-Ramírez, Ignacio Morrás, María Carnevali, Lorena Jiménez-Ibañez, Manuel Rubio-Rivas, Francisco Arnalich, José Luis Beato, Daniel Monge, Uxua Asín, Carmen Suárez, Santiago Jesús Freire, Manuel Méndez-Bailón, Isabel Perales, José Loureiro-Amigo, Ana Belén Gómez-Belda, Paula María Pesqueira, Ricardo Gómez-Huelgas, Carmen Mella, Luis Felipe Díez-García, Joaquim Fernández-Sola, Ruth González-Ferrer, Marina Aroza, Juan Miguel Antón-Santos, Carlos Lumbreras Bermejo

**Affiliations:** 1 Infectious Diseases Unit, Internal Medicine Department, Hospital Universitario Puerta de Hierro-Majadahonda UAM, IDIPHSA, Maestro Rodrigo 2, 28222 Majadahonda, Madrid, Spain; 2 Preventive Medicine Department, Hospital Universitario Puerta de Hierro-Majadahonda UAM, IDIPHSA, Maestro Rodrigo 2, 28222 Majadahonda, Madrid, Spain; 3 Internal Medicine Department, Hospital Universitario Puerta de Hierro-Majadahonda UAM, IDIPHSA, Maestro Rodrigo 2, 28222 Majadahonda, Madrid, Spain; 4 Internal Medicine Department, 12 de Octubre University Hospital, Av. de Córdoba, s/n, 28041 Madrid, Spain; 5 Internal Medicine Department, Gregorio Marañon University Hospital, Dr. Esquerdo, 46, 28007 Madrid, Spain; 6 Internal Medicine Department, Bellvitge University Hospital, Carrer de La Feixa Llarga, s/n, 08907 L’Hospitalet de Llobregat, Barcelona, Spain; 7 Internal Medicine Department, La Paz University Hospital, Paseo de La Castellana, 261, 28046 Madrid, Spain; 8 Internal Medicine Department, Albacete University Hospital Complex, Hermanos Falco, 37, 02006 Albacete, Spain; 9 Internal Medicine Department, Segovia Hospital Complex, Luis Erik Clavería Neurólogo s/n, 40002 Segovia, Spain; 10 Internal Medicine Department, Miguel Servet Hospital, Paseo Isabel la Católica, 1-3, 50009 Zaragoza, Spain; 11 Internal Medicine Department, La Princesa University Hospital, Diego de León, 62, 28006 Madrid, Spain; 12 Internal Medicine Department, A Coruña University Hospital, Xubias de Arriba, 84, 15006 A Coruña, Spain; 13 Internal Medicine Department, San Carlos Clinical Hospital, Prof Martín Lagos, s/n, 28040 Madrid, Spain; 14 Internal Medicine Department, Infanta Sofía Hospital, Paseo de Europa, 34, 28703 San Sebastián de los Reyes, Madrid, Spain; 15 Internal Medicine Department, Moisès Broggi Hospital, Carrer de Jacint Verdaguer, 90, 08970 Sant Joan Despí, Barcelona, Spain; 16 Internal Medicine Department, Dr. Peset University Hospital, Av. de Gaspar Aguilar, 90, 46017 Valencia, Spain; 17 Internal Medicine Department, Santiago Clinical Hospital, Rúa da Choupana, s/n, 15706 Santiago de Compostela, A Coruña, Spain; 18 Internal Medicine Department, Regional University Hospital of Málaga, Biomedical Research Institute of Málaga (IBIMA), University of Málaga (UMA), Av. de Carlos Haya, 84, 29010 Málaga, Spain; 19 Internal Medicine Department, Hospital Architect Marcide-Novoa Santos, Rúa Pardo Bazán, s/n, 15404 Ferrol, A Coruña, Spain; 20 Internal Medicine Department, Torrecárdenas Hospital, Hermandad de Donantes de Sangre, s/n, 04009 Almería, Spain; 21 Internal Medicine Department, Clinic Barcelona Hospital, Villarroel, 170, 08036 Barcelona, Spain; 22 Internal Medicine Department, Tajo Hospital, Av. Amazonas Central, s/n, 28300 Aranjuez, Madrid, Spain; 23 Internal Medicine Department, Insular de Gran Canaria Hospital, Av. Marítima del Sur, s/n, 35016 Las Palmas de Gran Canaria, Las Palmas, Spain; 24 Internal Medicine Department, Infanta Cristina University Hospital, Av. 9 de Junio, 2, 28981 Parla, Madrid, Spain; 25 Infectious Diseases Unit, Hospital Universitario Puerte de Hierro-Majadahonda Majadahonda, Calle Maestro Rodrigo 2, 28222 Majadahonda, Madrid, Spain

**Keywords:** Infectious diseases, Respiratory tract diseases

## Abstract

To determine the proportion of patients with COVID-19 who were readmitted to the hospital and the most common causes and the factors associated with readmission. Multicenter nationwide cohort study in Spain. Patients included in the study were admitted to 147 hospitals from March 1 to April 30, 2020. Readmission was defined as a new hospital admission during the 30 days after discharge. Emergency department visits after discharge were not considered readmission. During the study period 8392 patients were admitted to hospitals participating in the SEMI-COVID-19 network. 298 patients (4.2%) out of 7137 patients were readmitted after being discharged. 1541 (17.7%) died during the index admission and 35 died during hospital readmission (11.7%, p = 0.007). The median time from discharge to readmission was 7 days (IQR 3–15 days). The most frequent causes of hospital readmission were worsening of previous pneumonia (54%), bacterial infection (13%), venous thromboembolism (5%), and heart failure (5%). Age [odds ratio (OR): 1.02; 95% confident interval (95% CI): 1.01–1.03], age-adjusted Charlson comorbidity index score (OR: 1.13; 95% CI: 1.06–1.21), chronic obstructive pulmonary disease (OR: 1.84; 95% CI: 1.26–2.69), asthma (OR: 1.52; 95% CI: 1.04–2.22), hemoglobin level at admission (OR: 0.92; 95% CI: 0.86–0.99), ground-glass opacification at admission (OR: 0.86; 95% CI:0.76–0.98) and glucocorticoid treatment (OR: 1.29; 95% CI: 1.00–1.66) were independently associated with hospital readmission. The rate of readmission after hospital discharge for COVID-19 was low. Advanced age and comorbidity were associated with increased risk of readmission.

## Introduction

The novel coronavirus disease 2019 (COVID-19) pandemic has dramatically impacted many hospitals around the world. For several weeks, the demand for hospital beds in Spain surpassed the capacity to admit patients, hindering the ability to treat other serious illnesses such as neoplasms or cardiovascular disease^1–3^. After the highest incidence of this infection during the months of March and April 2020, outbreaks of varying magnitudes are being observed in different regions throughout the world that could eventually compromise hospitals' capacity again^4^. Therefore, improving knowledge on the course of the disease could contribute to the appropriate use of the health resources available within the epidemiological framework.

The aforementioned potential shortage of hospital beds and the lack of firmly established discharge recommendations could result in the hasty, risky discharge of admitted patients. Conversely, physicians may act in an overly cautious manner, unnecessarily prolonging hospital stays. Although the evolution of microbiological tests after the acute phase of the disease [polymerase chain reaction (PCR) of nasal exudate)] has been the subject of several studies, the clinical characteristics of patients at increased risk of readmission have been analyzed in a limited number of studies^5–10^. Increased knowledge of the magnitude and characteristics of this issue could help in decision-making related to the initial hospital stay, time of discharge, and clinical follow-up after discharge.

The SEMI-COVID-19 Network has arisen as an initiative of the Spanish Society of Internal Medicine (SEMI, for its initials in Spanish) to improve the management of COVID-19. The main objective of the registry is to generate, in a short period of time, a large, multicenter cohort with detailed information on the epidemiology, clinical progress, and treatment received by patients^11^. Using this information, we aimed to determine the proportion of COVID-19 patients who were readmitted after discharge, the causes of readmission, and factors associated with this poor outcome.

## Methods

### Study design

This is an observational study base on the SEMI-COVID-19 Registry, which is a retrospective cohort comprising consecutive patients admitted in 147 hospitals in Spain from March 1, 2020 discharged with confirmed COVID-19 disease. The aim of the study was to analyze the clinical characteristics of patients with COVID-19 who were readmitted to the hospital during the first 30 days after being discharged. Patients included in the SEMI-COVID-19 Registry from March 1, 2020 to April 30, 2020 were included in this study, representing approximately 10% of the patients admitted in Spain during this time period^1^. Comparative group was constituted by patients who were discharged alive from the primary admission and were not readmitted during the 30 days following discharge. Patients who were attended in the emergency department after hospital discharge but that were not admitted, were not considered as readmitted patient.

### Study population and participants

All consecutive patients discharged after hospital admission with confirmed SARS-CoV-2 infection were eligible for inclusion in the SEMI-COVID-19 Registry^11^. COVID-19 was confirmed by a positive result on real-time polymerase chain reaction (RT-PCR) testing of a nasopharyngeal or sputum sample. Patients were treated at their attending physician’s discretion, according to local protocols and clinical judgement. Patients included in open-label clinical trials could be included in the registry, provided all information about treatment was available.

### Data collection

Data were entered into the database by retrospective review of medical records by resident or staff physicians of the internal medicine departments of the participating hospitals. An online electronic data capture system was developed, which includes a database manager along with procedures for the verification of data and contrasting of information against the original medical record in order to ensure the best possible quality of data collection. Patient identifiable data were dissociated and pseudonymized. Collected data and included epidemiological data, RT-PCR and serology data, medical and medication history, symptoms and physical examination findings at admission, laboratory and diagnostic imaging tests, treatment, complications during the hospitalization, and hospital readmission. Obesity was defined as a body mass index greater than 30 kg/m2. Acute cardiac injury was defined as the detection of acute myocardial infarction, heart failure, arrhythmia or myocarditis. Acute kidney injury was defined as a 50% increase in the baseline creatinine level or a creatinine level greater than 1.5 mg/dl if the previous value was unknown.

Immunocompromised patients included those with solid organ, hematopoietic stem cell transplantation, glucocorticoid treatment (equivalent dose of prednisone ≥ 15 mg/day) or immunosuppressive drugs. Patients with HIV were reported in a separate group. Reasons for readmission, including respiratory symptoms, venous or arterial thrombosis, exacerbation of chronic diseases, organ failure, and bacterial infection were registered. Information on readmissions and death after admission was collected by retrospective review of the clinical history and by telephone contact with the patient and family members one month after discharge. Telephone tracking was not possible in 294 patients, which accounted for 4% of the patients discharged.

### Data analysis

Participants’ demographic, clinical, epidemiological, laboratory, and diagnostic imaging data during the first and second hospital admissions obtained from the Registry data base were analyzed. Treatment received, complications, and clinical progress were also examined. Quantitative variables are expressed as median [interquartile range] or median [SD]. Categorical variables are expressed as absolute frequencies and percentages. The chi-square test and Fisher’s exact test were used to compare categorical variables while Student’s t-test and the Mann–Whitney U Test were used to compare continuous variables. Univariate and multivariate logistic regression models were created using Stata 14.0 software (Stata Corp., College Station, USA). All tests of significance were two-tailed and p values < 0.05 were considered statistically significant. A logistic regression was performed by grouping common variables with a p value < 0.1 and clinical relevance and a number of missings < 10%.

### Ethical aspects

Personal data is processed in strict compliance with Spanish Law 14/2007, of July 3, on Biomedical Research; Regulation (EU) 2016/679 of the European Parliament and of the Council of 27 April 2016 on the protection of natural persons with regard to the processing of personal data and on the free movement of such data, and repealing Directive 95/46/EC (General Data Protection Regulation); and Spanish Organic Law 3/2018, of December 5, on the Protection of Personal Data and the Guarantee of Digital Rights. The SEMI-COVID-19 Registry has been approved by the Provincial Research Ethics Committee of Málaga (Spain). We attempted to obtain informed consent from all patients. When this was not possible due to biosafety issues, informed consent was requested verbally and noted in the medical record. The STROBE statement guidelines were followed in the conduct and reporting of this study. The information contained in the database may be accessible after contacting the corresponding author and after its reasoned justification.

## Results

**Figure 1:**
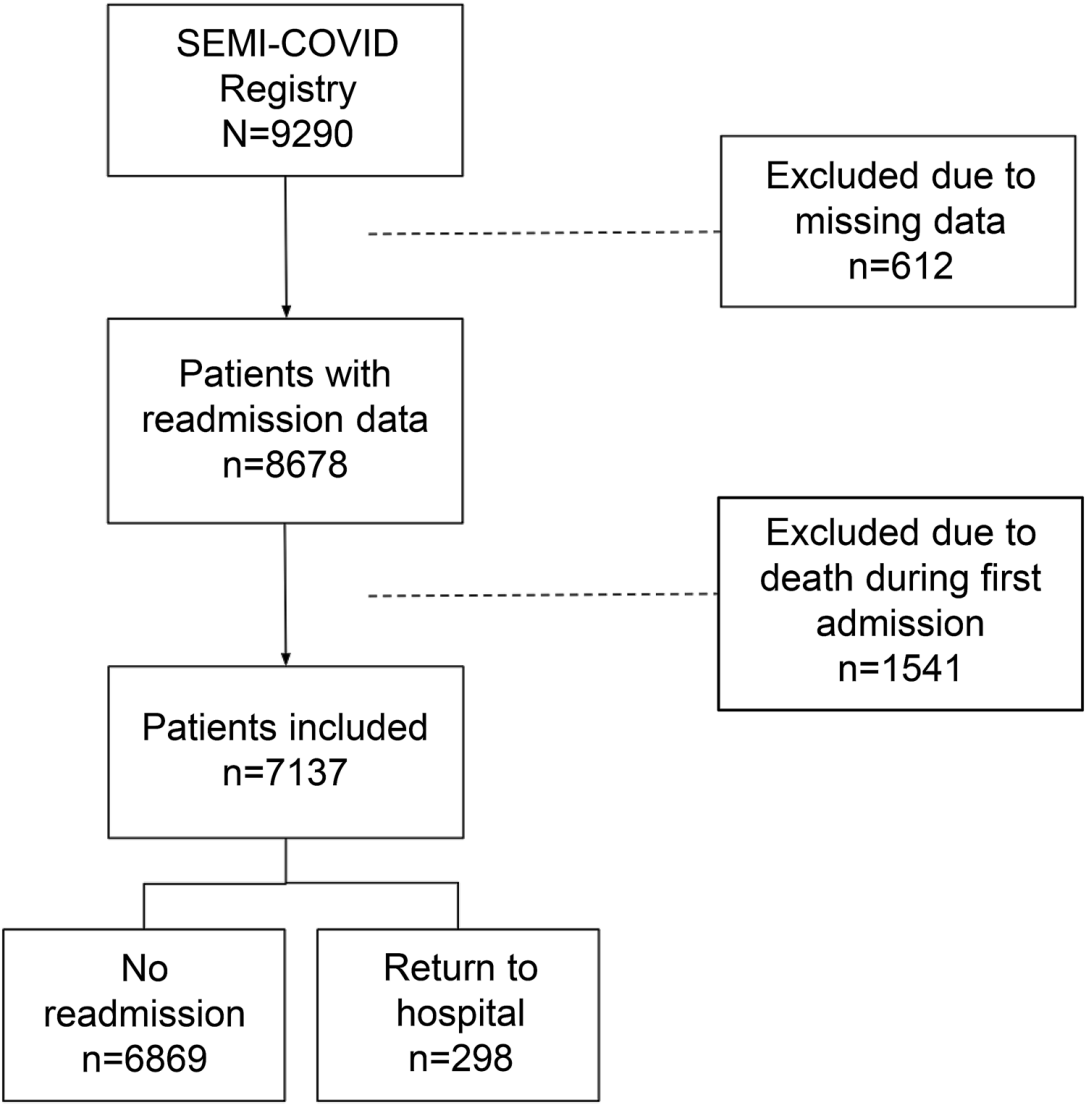
Flow chart of inclusion of patients with COVID-19 according to hospital readmission.

Over the course of the study period, 8678 patients were included in the registry. Of them, 7137 patients (82.2%) were discharged alive. In the following days, 298 patients (4.2%) were readmitted. The median time from discharge to readmission was 7 days (IQR 3–15). 1541 patients (17.7%) died during the index admission and 35 died during hospital readmission (11.7%, p = 0.007) (Fig. 1). Of the 6839 patients who were discharged alive and were not readmitted, 50 died during the first month after discharge (0.73%). Among the patients who received macrolides, azithromycin was the most frequently administered (99.1%).

### Clinical characteristics of the patients who are readmitted

Table 1 shows the clinical characteristic of patients who were readmitted compared to those who were not. The univariate analysis showed that readmitted patients were older (74 vs 65 years, p < 0.001) and had more severe comorbidity. The duration of symptoms before the index admission was shorter in patients who were subsequently readmitted (p < 0.001). A higher percentage of patients who were readmitted had received steroid treatment compared to those who were not readmitted (38.3% vs 29.7%, p = 0.002, Table 2). The proportion of patients who received prophylactic low-molecular-weight heparin (LMWH) during the primary admission and were readmitted for DVT (87.5%) was similar to patients who were readmitted for another reason (86.2%, p = 0.885) and to patients who were not readmitted (82.7%, p = 0.645).

**Table 1:** Clinical characteristics of patients with COVID-19 according to hospital readmission.

Characteristics	No readmission n = 6839	Missing	Readmission n = 298	Missing	p	Overall missing
**Demographic**						
Age (median, IQR)	65 (53–75)	4 (0,1%)	74 (60–83)	1 (0,3%)	< 0.001	5 (0.1%)
Obesity (n, %)	1297 (19.0)	640 (9.3%)	70 (23.5)	23 (7.7%)	0.054	663 (9.2%)
Age-adjusted Charlson Comorbidity Index (mean, SD)	3.0 (2.4)	231 (3.4%)	4.4 (2.7)	9 (3.0%)	< 0.001	240 (3.4%)
Male gender (n, %)	3849 (56.3)	0	173 (58.0)	0	0.562	0
Ethnicity (n, %)						
Caucasian	5878 (85.8)	0	259 (86.9)	0	0.594	0
Black	36 (0.5)		0 (0.0)			
Hispanic	684 (10.0)		29 (9.7)			
Asian	41 (0.6)		2 (0.7)			
Other	80 (1.3)		1 (0.3)			
Unknown	120 (1.8)		7 (2.4)			
**Comorbidity (n, %)**						
Hypertension	3081 (45.1)	3 (< 0.1%)	166 (55.7)	0	< 0.001	3 (< 0.1%)
Diabetes	1113 (16.3)	0	64 (21.1)	0	0.029	0
Cardiovascular disease	833 (12.2)	458 (6.7%)	72 (24.2)	19 (6.4%)	< 0.001	477 (6.6%)
Asthma	554 (8.1)	3 (< 0.1%)	37 (12.4)	0	0.008	3 (< 0.1%)
Chronic obstructive pulmonary disease	364 (5.3)	0	42 (14.1)	0	< 0.001	0
Cerebrovascular disease	349 (5.1)	265 (3.9%)	25 (8.4)	5 (1.7%)	0.012	270 (3.8%)
HIV infection	58 (0.8)	30 (0.4%)	1 (0.3)	0	0.341	30 (0.4%)
Solid tumor	467 (6.8)	29 (0.4%)	29 (9.7)	0	0.049	29 (0.4%)
Hematologic malignancies	112 (1.6)	0	5 (1.7)	0	0.645	0
Immunosuppression	225 (3.3)	26 (0.4%)	7 (2.4)	0	0.394	26 (0.4%)
End-stage kidney failure (dialysis)	45 (0.7)	52 (0.7%)	6 (2.0)	2 (0.7%)	0.001	54 (0.7%)
Chronic hepatopathy	227 (3.3)	0	17 (5.7)	0	0.029	0
Dementia	427 (6.2)	20 (0.3%)	39 (13.1)	0	< 0.001	20 (0.3%)
**Hospital admission characteristics**						
Acquisition (n,%)						
Community	6469 (94.6)	0	269 (90.3)	0	0.001	0
Long-term care facility	370 (5.4)	0	29 (9.7)	0	0.001	0
Duration of symptoms (median, IQR)	7 (4–10)	84 (1.2%)	5 (3–8)	3 (1.0%)	< 0.001	87 (1.2%)
Length of index hospital stay	9 (6–14)	5 (< 0.1%)	9 (6–15)	0	0.461	5 (< 0.1%)
ICU admission	390 (5.7)	0	12 (4.0)	0	0.219	0
Systolic blood pressure, mm Hg (mean, SD)	129.0 (20.3)	356 (5.2%)	131.6 (23.9)	13 (4.4%)	0.038	369 (5.2%)
Diastolic blood pressure, mm Hg (mean, SD)	75.0 (12.7)	360 (5.3%)	72.9 (13.6)	15 (5.0%)	0.009	375 (5.2%)
Temperature, °C (mean, SD)	37.1 (1.0)	246 (3.6%)	37.1 (0.9)	8 (2.7%)	0.660	242
Radiological pattern (n,%)*						
Ground-glass opacity	4236 (61.9)	75 (1.1%)	149 (50.0)	5 (1.7%)	< 0.001	80 (1.1%)
Pleural effusion	218 (3.2)	76 (1.1%)	15 (5.0)	7 (2.3%)	0.086	83 (1.2%)
Consolidation	3228 (47.2)	87 (1.3%)	112 (37.6)	5 (1.7%)	0.006	92 (1.3%)

*A patient could have several radiological patterns.

**Table 2:** Treatment, complications, and progress of patients with COVID-19 depending on hospital readmission.

Treatments	No readmission n = 6839	Missing	Readmission n = 298	Missing	p	Overall missing
Duration of invasive ventilation (median, IQR)	10 (7–15)	28 (0.4%)	8 (6–16)	1 (0.3%)	0.966	29 (0.4%)
Duration of noninvasive ventilation (median, IQR)	4 (2–8)	32 (0.5%)	4 (3–8)	1 (0.3%)	0.612	33 (0.5%)
Glucocorticoid treatment (n, %)	2034 (29.7)	53 (0.8%)	114 (38.3)	1 (0.3%)	0.002	54 (0.8%)
LMWH, prophylactic dose, during admission (n,%)	5658 (82.7)	0.9% (65)	246 (82.6)	5 (1.7%)	0.791	70 (0.9%)
Remdesivir (n, %)	31 (0.5)	86 (1.3%)	1 (0.3)	3 (1.0%)	0.611	89 (1.2%)
Interferon (n, %)	784 (11.5)	75 (1.0%)	32 (10.7)	0	0.674	75 (1.0%)
Lopinavir/Ritonavir (n, %)	4533 (66.3)	32 (0.5%)	163 (54.7)	0	< 0.001	32 (0.5%)
Macrolide (n, %)^1^	4291 (62.7)	44 (0.6%)	153 (51.3)	1 (0.3%)	< 0.001	45 (0.6%)
Hydroxychloroquine (n, %)	6128 (89.6)	22 (0.3%)	257 (86.2)	0	0.075	22 (0.3%)
Chloroquine (n, %)	256 (3.7)	56 (0.8%)	10 (3.4)	1 (0.3%)	0.722	57 (0.8%)
Tocilizumab (n, %)	577 (8.4)	55 (0.8%)	19 (6.4)	0	0.240	55 (0.8%)
**Complications (n, %)**						
Bacterial pneumonia	510 (7.5)	17 (0.2%)	31 (10.4)	0	0.062	17 (0.2%)
ARDS	1481 (21.7)	28 (0.4%)	59 (19.8)	2 (0.7%)	0.435	30 (0.4%)
Acute kidney injury	539 (7.9)	4 (< 0.1%)	40 (13.4)	0	< 0.001	4 (< 0.1%)
Acute cardiac injury	220 (3.2)	17 (0.2%)	39 (13.1)	0	< 0.001	17 (0.2%)
Stroke	21 (0.3)	16 (0.2%)	1 (0.3)	0	1.000	16 (0.2%)
Sepsis	136 (2.0)	4 (< 0.1%)	6 (2.0)	0	1.000	4 (< 0.1%)
Shock	82 (1.2)	39 (0.6%)	5 (1.7)	1 (0.3%)	0.414	40 (0.6%)
MOF	37 (0.5)	21 (0.3%)	4 (1.3)	0	< 0.001	21 (0.3%)

LMWH: Low-molecular-weight heparin, ARDS: acute respiratory distress syndrome, MOF: multiple organ failure. ^1^98.2% azithromycin, 1.2% other macrolides. *A patient could have several radiological patterns.

Age, Charlson Comorbidity Index score, diabetes, chronic obstructive pulmonary disease (COPD), asthma, solid neoplasia, hypertension, dementia, duration of symptoms before admission, hemoglobin level and platelets count at admission, ground-glass infiltrate at admission, acute cardiac injury, acute renal failure and steroid treatment during admission were included in the final model (Table 3). Age [odds ratio (OR) per one-year increase: 1.02; 95% confident interval (95% CI): 1.01–1.03], age-adjusted Charlson comorbidity index score (OR: 1.13; 95% CI: 1.06–1.21), COPD (OR: 1.84; 95% CI: 1.26–2.69), asthma (OR: 1.52; 95% CI: 1.04–2.22), hemoglobin level at admission (OR: 0.92; 95% CI: 0.86–0.99), ground-glass opacification at admission (OR: 0.86; 95% CI:0.76–0.98) and glucocorticoid treatment (OR: 1.29; 95% CI: 1.00–1.66) were independently associated with hospital readmission. The C-statistic of the model was 0.661.

**Table 3:** Final multivariate analysis of variables related to risk of readmission in patients with COVID-19.

Variable	adjusted OR	95%CI	p
Age ^1^	1.02	1.01–1.03	< 0.001
Age-adjusted Charlson Comorbidity Index score	1.13	1.06–1.21	0.001
Diabetes mellitus	1.05	0.74–1.47	0.796
Chronic obstructive pulmonary disease	1.84	1.26–2.69	0.002
Asthma	1.52	1.04–2.22	0.031
Solid neoplasm	0.70	0.42–1.18	0.186
Hypertension	0.88	0.67–1.16	0.251
Dementia	1.18	0.79–1.77	0.408
Duration of symptoms before admission	1.00	0.99–1.00	0.909
Hemoglobin level at admission	0.92	0.86–0.99	0.028
Platelets count at admission	1.00	1.00–1.00	0.175
Ground-glass opacification at admission	0.86	0.76–0.98	0.026
Acute cardiac injury^2^	1.23	0.74–2.00	0.416
Acute kidney failure	1.23	0.85–1.78	0.269
Glucocorticoid treatment	1.29	1.00–1.66	0.049

^1^Per one-year increase. ^2^ Acute cardiac injury: acute myocardial infarction, heart failure, arrhythmia or myocarditis.

The characteristics of patients who were not readmitted versus those who were readmitted because of worsening of SARS-CoV-2 pneumonia are shown in the supplementary material. Most of the variables associated with all-cause readmission were also related to readmission for pneumonia (Tables 1, 2, 4 and Table 3S in supplementary material). Patients who were readmitted for pneumonia had a lower prescription of prophylactic heparin at discharge. The rate of readmission for pneumonia was lower in patients who had been admitted to the ICU during the index admission (2%, 3 patients) than in those who had not been admitted to the ICU (5.7%, 390 patients; p = 0.041). (Table 3S in supplementary material). The specific multivariate analysis aggregated by type of variable is presented in the supplementary material.

**Table 4:** Analytical results of patients with COVID-19 according to hospital readmission.

	Cohort n = 6839	Missing	Readmission n = 298	Missing	p	Overall Missing
**Sample at admission**^1^						
Oxygen saturation, %	94.1 (4.3)	196 (2.9%)	93.9 (4.3)	8 (2.7%)	0.533	204 (2.9%)
Hemoglobin, g/dL	14.0 (1.7)	27 (0.4%)	13.3 (2.0)	0	< 0.001	27 (0.4%)
Platelets × 10^6^/L	204,993 (87,595.1)	35 (0.5%)	194,500 (79,822.9)	0	0.042	35 (0.5%)
White blood cell count/uL	5950 (4610–7860)	30 (0.4%)	6400 (4600–8400)	0	0.114	30 (0.4%)
Lymphocytes cel/uL	1000 (700–1309)	64 (0.9%)	924 (700–1300)	1 (0.3%)	0.228	53 (0.9%)
Neutrophils cel/uL	4200 (3025–6000)	83 (1.2%)	4600 (3100–6400)	2 (0.7%)	0.102	85 (1.2%)
Neutrophil-to-lymphocyte ratio	4.2 (2.7–7.0)	83 (1.2%)	4.6 (2.9–7.2)	2 (0.7%)	0.116	85 (1.2%)
Eosinophils × 10^6^/L	0.0 (0.0–20.0)	154 (2.2%)	0.0 (0.0–30.0)	3 (1.0%)	0.322	157 (2.2%)
C-Reactive Protein, mg/L	49.4 (15.7–107.9)	290 (4.2%)	47.5 (15.6–112.2)	8 (2.7%)	0.977	284 (4.2%)

^1^Sample at admission was obtained during the first 24 h of admission. Variables are expressed as median (interquartile range).

### Description of the indication for hospital readmission

Table 5 shows the reasons for the readmission. The most frequent causes were worsening of previous pneumonia (54%), bacterial infection (13%), venous thromboembolism (5%), and heart failure (5%). Acute kidney failure, neurological complications, and severe hemorrhage were less common as a cause of readmission.

**Table 5:** Cause of hospital readmission in 298 patients previously admitted due to COVID-19.

Condition	Hospital readmission (n = 298)
Pneumonia	158 (53.6)
Bacterial infection^1^	38 (12.8)
Venous thromboembolic disease	16 (5.4)
Heart failure^2^	16 (5.4)
Acute kidney failure	13 (4.4)
Encephalopathy or delirium	7 (2.3)
Chronic lung disease exacerbation	7 (2.3)
Severe hemorrhage	6 (2)
Home isolation impossibility	5 (1.7)
Generalized exanthema	4 (1.3)
Ischemic stroke	4 (1.3)
Acute hepatitis	4 (1.3)
Bone fracture	4 (1.3)
Social problem	4 (1.3)
Other conditions ^3^	19 (6.4)

^1^Respiratory (18 patients), urinary tract (9 patients), intra-abdominal (6 patients), acute gastroenteritis (2 patients), *Clostridioides difficile* colitis (1 patient), surgical wound (1 patient). ^2^ Five cases due to arrhythmia and one case due to acute myocardial infarction. ^3^ Psychosis (3 patients), acute pancreatitis (3 patients), vasculitis (3), diabetes with hyperosmolar state (2 patient), anemia (2 patients), viral syndrome due to Cytomegalovirus (1 patient), *Pneumocystis jirovecii* pneumonia, Guillain-Barré polyradiculopathy (1 patient), pneumothorax (1 patient), generalized seizure (1 patient), adenocarcinoma of the pancreas (1 patient). Seven patients presented more than one cause for hospital admission.

## Discussion

We present the first multicenter study on hospital readmissions of COVID-19 patients carried out in Spain. The main finding was that the rate of hospital readmission was relatively low with half of the readmissions occurring during the first week after discharge from the hospital. Most of them were due to respiratory worsening. Age, comorbidity (especially, asthma and COPD) were associated with an increased risk of readmission. Patients with higher hemoglobin levels and ground glass opacification at admission had a lower risk of readmission.

Our readmission rate was similar to those found by other investigators^7,10,12^, but lower than those observed in other studies^6,9,13,14^. The higher readmission rate observed in some studies can be related to the longer follow-up period in some of them^6,14^ and to the higher incidence of diseases such as obesity, hypertension, diabetes, asthma and chronic renal failure^6,9,14^. On the other hand, we cannot rule out that the severity of infection in the patients included in the study was lower than in other series, considering the uncertainties in clinical management, which were more prominent during the first wave of the disease. Our results indicate that, after an initial clinical improvement, a small but significant number of patients worsen and require readmission to hospital. The readmission rate in the present study appears to be lower than that observed in patients admitted for other reasons to internal medicine wards in Spain. This observation could be related to the older age and higher comorbidity of patients hospitalized for non-COVID-19-related reasons in our country^15^.

Although the average hospital occupancy per COVID-19 in Spain was around 10% during the first wave, it does not appear that there were many precipitous discharges, in view of the modest rate of readmissions^1,16^. The criteria usually followed to evaluate hospital discharge in patients with respiratory infection, such as disappearance of fever and improvement of respiratory failure, may be effective parameters for discharge ensuring a low readmission rate. We have the impression that the recommendations established in China at the beginning of the pandemic could be overly conservative (no fever for 3 days in addition to clinical, analytical, and radiological improvement) and could lead to an unnecessary prolongation of the hospital stay^5^.

Another characteristic of readmissions is their lower mortality compared to the index admission^6,13^. It cannot be ruled out that this finding could be influenced by the possible death of the most fragile patients during the index admission. The differences detected in the mortality of hospital readmissions in patients with COVID-19 between different hospitals could be justified by the degree of difficulty of admission of non-critical patients, which is related to the pressure on the healthcare system^10^.

### Clinical characteristics of the patients who are readmitted

Older age and comorbidity were associated with an increased risk of readmission, as has been reported in previous studies^5,17^. Both factors often predispose patients to complications that may require readmission^6,7^. Not unexpectedly, readmissions for an infection that primarily affects the lungs are more frequent in patients with chronic lung disease such as asthma or COPD^7,18,19^. Adjusting the treatment of chronic lung disease (corticosteroids, bronchodilators or oxygen flow) appropriately and determining the frequency of contacts with their physicians could be beneficial. Dementia was associated with an increased risk of readmission in the univariate analysis. Interestingly, patients transferred to a skilled nursing facility also showed an increased risk of readmission in previous studies^6^. The prevention and treatment of delirium during admission to reduce the risk of readmission is also one of the aspects to be considered^17^.

The duration of symptoms in patients who were readmitted (median 5 days) was shorter than in those who were not readmitted (IQR) (7 days, < 0.001, Table 2). The prolonged clinical course with frequent worsening that characterizes moderate and severe forms of the disease suggests that the time of onset of the patient's symptoms should be taken into account when considering hospital discharge^20,21^. A shorter duration of index admission has been associated with a higher risk of readmission^6,7^; a result that has not been observed in our series. Admission to the ICU has also been associated with a lower risk of readmission due to SARS-CoV_2 pneumonia in our patients, which may be related to a longer hospital stay of index admission and possible better medical care^6^.

Serum hypoalbuminemia at admission has been associated with a worse prognosis including the risk of hospital readmission^22^. Yeo et al., observed a direct correlation between the peak creatinine concentration during admission and the risk of readmission^10^. In our study, acute renal failure was associated with an increased risk of readmission but only in the univariate analysis. Hypoalbuminemia and renal dysfunction could be markers of more severe disease and multiorgan failure increasing the risk of readmission. The presence of ground-glass opacities (GGO) showed to be a protective factor for hospital readmission. This result may be explained by the fact that the presence of ground-glass opacities, both in unilateral and bilateral involvement, is associated with an early stage of the disease, which could be related to the risk of hospital readmission^23^. The efficacy of the combination of certain clinical variables and analytical markers in the prediction of hospital readmission could be a line of research that could improve our knowledge of the evolution of this disease^16,24^.

In our study the association between glucocorticoid treatment and hospital readmission was remarkable. Since there was no generalized prescription of corticosteroids during the first wave, we cannot rule out that this treatment was a surrogate marker of greater pulmonary involvement predisposing to hospital readmission^25^. In addition, a positive association was found in the univariant analysis between macrolide treatment and a reduction in risk of hospital readmission. These results are surprising considering the results of several clinical studies that examined the role of azithromycin (administered together with hydroxychloroquine) in the prognosis of COVID-19 and found no benefit in terms of mortality or significant clinical improvement^26,27^. Macrolide treatment was not included in the multivariate analysis because we are not sure that its association with the risk of readmission was due to variables not analyzed.

### Description of the cause for admission at initial admission and readmission

As in other similar studies, worsening SARS-CoV-2 pneumonia, bacterial infection, venous thromboembolism, heart failure, acute renal failure, and neurological or psychiatric conditions were common causes of hospital readmission^5,7,13^. The administration of more effective and less toxic treatments, determining the dose and rate of decrease of steroids, and the development of prognostic scores could lead to fewer or milder exacerbations of SARS-CoV-2 pneumonia^28,29^.

Bacterial infection was a common cause of readmission. Early removal of invasive devices and close monitoring of infection symptoms are important measures, given the late onset of some nosocomial infections in these patients and the masking effect of steroids or interleukin inhibitors on clinical presentation^8,30^.

Venous thromboembolism was also a relatively frequent cause of readmission that usually appeared in the four weeks following the initial hospital admission^31^. This disease is related to coagulopathy, viral endothelial damage due to COVID-19, and immobilization during hospitalization^31^. Although our study is limited in its ability to evaluate this disease, use of LMWH was not associated with a lower risk of readmission due to DVT^32^. Likewise, there is still a need to better define the risk estimate and the dose and duration of prophylactic LMWH in COVID-19 patients^31,32^.

Worsening of cardiac, digestive, endocrine or urinary diseases due to COVID-19 their sequelae appear to be frequent reasons for readmission in our and other studies^6^. Therapy during index admission such as the infusion of IV fluids, diuretics, nephrotoxic drugs, should be given with great caution, given its possible relationship with heart and kidney failure that could precipitate a readmission^33^. Preventive strategies according to comorbidity of the patient may reduce the risk of organ failure and readmission in covid-19 patients. A close contact with the patient, provided with an app., a thermometer and a pulse oximeter, after discharge has resulted in a lower risk of readmission^34^.

### Limitations

Several limitations of the study should be pointed out. First, as a multicenter study, it is likely that the discharge criteria at each of the participating hospitals differed, so quite different patients may have been analyzed all together. Second, relevant variables such as persistence of fever in the final hours before discharge were not collected and as such, we are unable to determine their relationship to readmission risk or compare them to other variables. Third, the learning curve for this new disease and changes in the demand for hospital admissions during the course of the epidemic may have increased disparity in patient management. Fourth, since the patients included in this study corresponded to the months of March and April 2020, the COVID-19 treatment they received was different from that subsequently recommended. Specifically, there has been a decrease in the use of drugs that we now know do not reduce mortality (hydroxychloroquine, azithromycin, lopinavir-ritonavir, interferon) and an increase in the use of steroids and interleukin inhibitors. Fifth, another limitation is the inability to analyze whether some patients remained hospitalized longer solely due to social and epidemiological reasons (i.e. being unable to take precautions to reduce transmission of the infection at home). Sixth, since we have not analyzed the number of patients who returned to the emergency department with persistent symptoms but were not readmitted during the peak of the pandemic, we must acknowledge some uncertainty in the assertion that the reduced proportion of readmissions was due to a favorable clinical outcome. Lastly, some variables were missing results for a large number of patients, especially in the case of procalcitonin, interleukin-6, D-dimer, lactate dehydrogenase and ferritin. In any case, only variables with a number of failures lower than 10% were included in the multivariate analysis. Despite this, we believe that the information obtained is useful for the management of patients admitted with COVID-19.

## Conclusions

In conclusion, we observed a low readmission rate after discharge from the hospital for COVID-19. Worsening of previous pneumonia, bacterial infection, venous thromboembolism, and heart failure were common causes of hospital readmission. Advanced age and comorbidity were associated with an increased risk of readmission. Our study could help to recognize patients at high risk of readmission, which would allow the establishment of strategies during the index admission and after discharge to reduce the frequency of these episodes.

## Supplementary Information

Supplementary Information.

## References

[1] Working group for the surveillance and control of COVID-19 in Spain; Members of the Working group for the surveillance and control of COVID-19 in Spain. The first wave of the COVID-19 pandemic in Spain: characterisation of cases and risk factors for severe outcomes, as at 27 April 2020. *Euro Surveill* **25**, 2001431 (2020). 10.2807/1560-7917.ES.2020.25.50.2001431 PMC7812423 33334400

[2] Dafer RM, Osteraas ND, Biller J (2020). Acute stroke care in the Coronavirus Disease 2019 Pandemic. J. Stroke Cerebrovasc. Dis..

[3] Al-Shamsi HO, Alhazzani W, Alhuraiji A (2020). A practical approach to the management of cancer patients during the novel Coronavirus Disease 2019 (COVID-19) Pandemic: An international collaborative group. Oncologist.

[4] Eurosurveillance editorial team (2020). Rapid risk assessment from ECDC: Resurgence of reported cases of COVID-19 in the EU/EEA, the UK and EU candidate and potential candidate countries. Euro Surveill..

[5] Richardson S, Hirsch JS, Narasimhan M (2020). Presenting characteristics, comorbidities, and outcomes among 5700 patients hospitalized With COVID-19 in the New York City Area. JAMA.

[6] Lavery AM, Preston LE, Ko JY (2020). Characteristics of hospitalized COVID-19 patients discharged and experiencing same-hospital readmission - United States, March-August 2020. Morb. Mortal Wkly. Rep..

[7] Somani SS, Richter F, Fuster V (2020). Characterization of patients who return to hospital following discharge from hospitalization for COVID-19. J. Gen. Intern. Med..

[8] Kang H, Wang Y, Tong Z, Liu X (2020). Retest positive for SARS-CoV-2 RNA of "recovered" patients with COVID-19: Persistence, sampling issues, or re-infection?. J. Med. Virol..

[9] Verma AA, Hora T, Jung HY (2021). Characteristics and outcomes of hospital admissions for COVID-19 and influenza in the Toronto area. CMAJ.

[10] Yeo, I., Baek, S., Kim, J., et al. Assessment of thirty-day readmission rate, timing, causes and predictors after hospitalization with COVID-19. *J Intern Med* (2021) [Epub ahead of print]. 10.1111/joim.13241 PMC8013754 33452824

[11] Casas-Rojo JM, Antón-Santos JM, Millán-Núñez-Cortés J (2020). Clinical characteristics of patients hospitalized with COVID-19 in Spain: Results from the SEMI-COVID-19 Registry. Rev. Clin. Esp. (English Edition).

[12] Wang X, Xu H, Jiang H (2020). The clinical features and outcomes of discharged Coronavirus disease 2019 Patients. A prospective cohort study. QJM.

[13] Loerinc LB, Scheel AM, Evans ST (2021). Discharge characteristics and care transitions of hospitalized patients with COVID-19. Healthcare (Amst).

[14] Chopra V, Flanders SA, O'Malley M, Malani AN, Prescott HC (2021). Sixty-day outcomes among patients hospitalized With COVID-19. Ann. Intern. Med..

[15] Zapatero A, Barba R, Marco J (2012). Predictive model of readmission to internal medicine wards. Eur. J. Intern. Med..

[16] Bernal-Delgado E, García-Armesto S, Oliva J, Sánchez-Martínez FI, Repullo JR, Peña-Longobardo LM, Ridao-López M, Hernández-Quevedo C (2018). Spain: Health system review. Health Syst. Transit..

[17] LaHue SC, Douglas VC, Kuo T (2019). Association between inpatient delirium and hospital readmission in patients ≥ 65 years of age: A retrospective cohort study. J Hosp Med.

[18] Satici, C., Demirkol, M. A., Sargin Altunok, E., et al. Performance of pneumonia severity index and CURB-65 in predicting 30-day mortality in patients with COVID-19. *Int. J. Infect. Dis*. **98**, 84–89. (2020). 10.1016/j.ijid.2020.06.038. Epub ahead of print 10.1016/j.ijid.2020.06.038 PMC7293841 32553714

[19] Shin B, Kim SH, Yong SJ, Lee WY, Park S, Lee SJ, Lee SJ, Lee MK (2019). Early readmission and mortality in acute exacerbation of chronic obstructive pulmonary disease with community-acquired pneumonia. Chron. Respir. Dis..

[20] Wang QJ, Yao YZ, Song JS (2020). Kinetic changes in virology, specific antibody response and imaging during the clinical course of COVID-19: A descriptive study. BMC Infect. Dis..

[21] Zhou F, Yu T, Du R, Fan G, Liu Y, Liu Z, Xiang J, Wang Y, Song B, Gu X, Guan L, Wei Y, Li H, Wu X, Xu J, Tu S, Zhang Y, Chen H, Cao B (2020). Clinical course and risk factors for mortality of adult inpatients with COVID-19 in Wuhan, China: A retrospective cohort study. Lancet.

[22] Kheir M, Saleem F, Wang C, Mann A, Chua J (2021). Higher albumin levels on admission predict better prognosis in patients with confirmed COVID-19. PLoS ONE.

[23] Martínez Chamorro E, Díez Tascón A, Ibáñez Sanz L, Ossaba Vélez S, Borruel NS (2021). Radiologic diagnosis of patients with COVID-19. Radiologia.

[24] Liu YP, Li GM, He J (2020). Combined use of the neutrophil-to-lymphocyte ratio and CRP to predict 7-day disease severity in 84 hospitalized patients with COVID-19 pneumonia: a retrospective cohort study. Ann. Transl. Med..

[25] Soriano V, Ganado-Pinilla P, Sanchez-Santos M (2021). Main differences between the first and second waves of COVID-19 in Madrid, Spain. Int. J. Infect. Dis..

[26] RECOVERY Collaborative Group (2021). Azithromycin in patients admitted to hospital with COVID-19 (RECOVERY): A randomised, controlled, open-label, platform trial. Lancet.

[27] Cavalcanti AB, Zampieri FG, Rosa RG (2020). Hydroxychloroquine with or without Azithromycin in Mild-to-Moderate Covid-19. N. Engl. J. Med..

[28] Wendel Garcia PD, Thierry Fumeaux T, Guerci P (2020). Prognostic factors associated with mortality risk and disease progression in 639 critically ill patients with COVID-19 in Europe: Initial report of the international RISC-19-ICU prospective observational cohort. EClinicalMedicine.

[29] Dong YM, Sun J, Li YX (2021). Development and Validation of a Nomogram for Assessing Survival in Patients with COVID-19 Pneumonia. Clin. Infect. Dis..

[30] He Y, Li W, Wang Z, Chen H, Tian L, Liu D (2020). Nosocomial infection among patients with COVID-19: A retrospective data analysis of 918 cases from a single center in Wuhan. Infect. Control Hosp. Epidemiol..

[31] Klok FA, Kruip MJHA, van der Meer NJM (2020). Confirmation of the high cumulative incidence of thrombotic complications in critically ill ICU patients with COVID-19: An updated analysis. Thromb.

[32] Poggiali E, Bastoni D, Ioannilli E, Vercelli A, Magnacavallo A (2020). Deep Vein Thrombosis and Pulmonary Embolism: Two Complications of COVID-19 Pneumonia?. Eur. J. Case Rep. Intern. Med..

[33] Patel P, Sengupta N (2020). PPIs and beyond: A framework for managing anticoagulation-related gastrointestinal bleeding in the era of COVID-19. Dig Dis Sci..

[34] Gordon WJ, Henderson D, DeSharone A (2020). Remote patient monitoring program for hospital discharged COVID-19 Patients. Appl. Clin. Inform..

