# Supplementary material for: Frequency, risk factors, and outcomes of hospital readmissions of COVID-19 patients
Source: Sci Rep. 2021 Jul 2;11:13733. doi: 10.1038/s41598-021-93076-0 (PMC8253752; doi:10.1038/s41598-021-93076-0)
Supplement: Supplementary file 1 — Supplementary Information. [file 41598_2021_93076_MOESM1_ESM.docx]

**Supplementary material**

Title: Frequency, risk factors, and outcomes of hospital readmissions of COVID-19 patients: A nationwide multicenter study in Spain

Running title: Hospital readmissions of COVID-19 patients

AUTHORS:

Antonio Ramos-Martínez MD PhD. aramos220@gmail.com. Infectious Diseases Unit, Internal Medicine Department, Hospital Universitario Puerta de Hierro-Majadahonda UAM, IDIPHSA. Maestro Rodrigo 2, 28222. Majadahonda, (Madrid), Spain

Lina Marcela Parra-Ramírez MD. licela15@gmail.com. Preventive Medicine Department. Hospital Universitario Puerta de Hierro-Majadahonda UAM, IDIPHSA. Maestro Rodrigo 2, 28222. Majadahonda, (Madrid), Spain

Ignacio Morrás MD. i.morrastorre@gmail.com. Internal Medicine Department. Hospital Universitario Puerta de Hierro-Majadahonda UAM, IDIPHSA. Maestro Rodrigo 2, 28222. Majadahonda, (Madrid), Spain

María Carnevali MD. macarfri@gmail.com. Internal Medicine Department, 12 de Octubre University Hospital. Av. de Córdoba, s/n, 28041. Madrid. Spain

Lorena Jiménez-Ibañez MD PhD. ljimenezi@salud.madrid.org. Internal Medicine Department, Gregorio Marañon University Hospital. Dr. Esquerdo, 46, 28007 Madrid.

Manuel Rubio-Rivas MD PhD. mrubio@bellvitgehospital.cat. Internal Medicine Department, Bellvitge University Hospital. Carrer de la Feixa Llarga, s/n, 08907 L'Hospitalet de Llobregat, Barcelona. Spain.

Francisco Arnalich MD PhD. farnalich@salud.madrid.org. Internal Medicine Department, La Paz University Hospital. Paseo de la Castellana, 261, 28046 Madrid Spain.

José Luis Beato MD. jlbeato@sescam.org. Internal Medicine Department, Albacete University Hospital Complex. Hermanos Falco, 37, 02006 Albacete. Spain

Daniel Monge MD. dmonge5@hotmail.com. Internal Medicine Department, Segovia Hospital Complex. Luis Erik Clavería Neurólogo s/n 40002 Segovia. Spain

Uxua Asín MD. insamper@gmail.com. Internal Medicine Department, Miguel Servet Hospital. Paseo Isabel la Católica, 1-3, 50009 Zaragoza. Spain

Carmen Suárez MD PhD. csuarezfe@gmail.com. Internal Medicine Department, La Princesa University Hospital. Diego de León, 62, 28006 Madrid. Spain

Santiago Jesús Freire MD. santiago.freire.castro@sergas.es. Internal Medicine Department, A Coruña University Hospital. Xubias de Arriba, 84, 15006 A Coruña. Spain

Manuel Méndez-Bailón MD. manuelmenba@hotmail.com. Internal Medicine Department, San Carlos Clinical Hospital. Prof Martín Lagos, s/n, 28040 Madrid. Spain

Isabel Perales MD. isabel.perales@salud.madrid.org. Internal Medicine Department, Infanta Sofía Hospital. Paseo de Europa, 34, 28703 San Sebastián de los Reyes, Madrid. Spain

José Loureiro MD. ahores@gmail.com. Internal Medicine Department, Moisès Broggi Hospital. Carrer de Jacint Verdaguer, 90, 08970 Sant Joan Despí, Barcelona. Spain

Ana Belén Gómez-Belda MD PhD. agomezbelda@gmail.com. Internal Medicine Department, Dr. Peset University Hospital. Av. de Gaspar Aguilar, 90, 46017 Valencia. Spain

Paula María Pesqueira MD. paulapesqueira@hotmail.com. Internal Medicine Department, Santiago Clinical Hospital. Rúa da Choupana, s/n, 15706 Santiago de Compostela, A Coruña. Spain

Ricardo Gómez-Huelgas MD PhD. ricardogomezhuelgas@hotmail.com. Internal Medicine Department, Regional University Hospital of Málaga, Biomedical Research Institute of Málaga (IBIMA), University of Málaga (UMA). Av. de Carlos Haya, 84, 29010 Málaga. Spain

Carmen Mella MD. mellacarmen@gmail.com. Internal Medicine Department, Ferrol University Hospital Complex. Rúa Pardo Bazán, s/n, 15404 Ferrol, A Coruña. Spain

Luis Felipe Díez-García MD. lfdiez@telefonica.net. Internal Medicine Department, Torrecárdenas Hospital. Hermandad de Donantes de Sangre, s/n, 04009 Almería. Spain

Joaquim Fernández-Sola MD. jfernand@clinic.cat. Internal Medicine Department, Clinic Barcelona Hospital. Villarroel, 170, 08036 Barcelona. Spain

Ruth González-Ferrer MD. ruthgferrer@gmail.com. Internal Medicine Department, Tajo Hospital. Av. Amazonas Central, s/n, 28300 Aranjuez, Madrid. Spain

Marina Aroza MD. marina.aroza.e@gmail.com. Internal Medicine Department, Insular de Gran Canaria Hospital. Av. Marítima del Sur, s/n, 35016 Las Palmas de Gran Canaria, Las Palmas. Spain

Juan Miguel Antón-Santos MD. juanmi.anton@gmail.com. Internal Medicine Department, Infanta Cristina University Hospital . Av. 9 de Junio, 2, 289 marina.aroza.e@gmail.com 81 Parla, Madrid. Spain

Carlos Lumbreras Bermejo MD PhD. clumbrerasb@gmail.com. Internal Medicine Department, 12 de Octubre University Hospital. Av. de Córdoba, s/n, 28041. Madrid. Spain

Table 1S. Multivariate analysis of demographic and comorbidity variables related to risk of readmission

| **Characteristics** | **adjusted OR** | **95%CI** | **p** |
| --- | --- | --- | --- |
| Age | 1.01 | 1.00-1.02 | 0.049 |
| Obesity | 1.36 | 1.01-1.81 | 0.041 |
| Age-adjusted Charlson Comorbidity Index score | 1.16 | 1.08-1.24 | 0.000 |
| Acquisition | 1.00 | 0.78-1.27 | 0.972 |
| Hypertension | 0.89 | 0.67-1.18 | 0.422 |
| Diabetes | 0.86 | 0.61-1.21 | 0.382 |
| Chronic obstructive pulmonary disease | 1.87 | 1.27-2.74 | 0.001 |
| Asthma | 1.47 | 1.00-2.17 | 0.048 |
| Dementia | 1.34 | 0.87-2.06 | 0.190 |
| Dialysis | 1.34 | 0.55-3.30 | 0.522 |

Table 2S. Multivariate analysis of clinical and radiological presentation and therapy related to risk of readmission *

|  | **adjusted OR** | **95%CI** | **p** |
| --- | --- | --- | --- |
| Duration of symptoms | 1.00 | 0.99-1.00 | 0.917 |
| Ground-glass opacity | 0.86 | 0.75-0.97 | 0.023 |
| Condensation | 0.87 | 0.75-1.01 | 0.074 |
| Pleural effusion | 1.14 | 0.76-1.73 | 0.502 |
| Glucocorticoid treatment | 1.31 | 1.02-1.70 | 0.035 |
| Lopinavir/ritonavir treatment | 0.86 | 0.66-1.11 | 0.260 |
| Macrolide treatment | 0.62 | 0.49-0.80 | <0.001 |

**Adjusted by age, obesity, and age-adjusted Charlson Comorbidity Index score*

Table 3S. Clinical and analytical characteristics of patients with COVID-19 according to hospital readmission due to worsening pneumonia due to SARS-CoV-2.

| **Characteristics** | **Cohort n=6839** | **Readmission due to pneumonia**  **n=158** | **p** | **Missings** |
| --- | --- | --- | --- | --- |
| **Demographic** |  |  |  |  |
| Age (median, IQR) | 65 (53-75) | 73 (59-82) | <0.001 | 5 (0.1%) |
| Obesity (n, %) | 1297 (19.0) | 32 (20.3) | 0.681 | 663 (9.2%) |
| Age-adjusted Charlson Comorbidity Index score (mean, SD) | 3.0 (2.4) | 4.4 (2.8) | <0.001 | 240 (3.4%) |
| Male (n, %) | 3849 (56.3) | 97 (61.4) | 0.201 |  |
| **Comorbidities (n, %)** |  |  |  |  |
| Hypertension | 3081 (45.1) | 81 (51.3) | 0.122 | 3 (<0.1%) |
| Diabetes | 1120 (16.4) | 31 (19.6) | 0.284 | 0 |
| Cardiovascular disease | 833 (12.2) | 35 (22.2) | <0.001 | 477 (6.6%) |
| Asthma | 554 (8.1) | 20 (12.7) | 0.037 | 3 (<0.1%) |
| Chronic obstructive pulmonary disease | 364 (5.3) | 25 (15.8) | <0.001 | 0 |
| Cerebrovascular disease | 349 (5.1) | 14 (8.9) | 0.033 | 270 (3.8%) |
| HIV infection | 58 (0.8) | 1 (0.6) | 0.779 | 30 (0.4%) |
| Solid tumor | 467 (6.8) | 17 (10.8) | 0.049 | 29 (0.4%) |
| Hematologic malignancies | 112 (1.6) | 5 (3.2) | 0.117 | 0 |
| Immunosuppression | 225 (3.3) | 4 (2.5) | 0.577 | 26 (0.4%) |
| Dialysis | 45 (0.7) | 3 (1.9) | 0.079 | 54 (0.7%) |
| Chronic hepatopathy | 227 (3.3) | 12 (7.6) | 0.003 | 0 |
| Dementia | 427 (6.2) | 18 (11.4) | 0.008 | 20 (0.3%) |
| **Hospital admission characteristics** | |  |  |  |
| Acquisition (n,%) |  |  |  |  |
| Community | 6469 (94.6) | 143 (90.5) | 0.025 | 0 |
| Long-term care facility | 370 (5.4) | 15 (9.5) |  | 0 |
| Duration of symptoms (median, IQR) | 7 (4-10) | 6 (3-8) | 0.85 | 87 (1.2%) |
| ICU admission (n, %) | 390 (5.7) | 3 (2.0) | 0.041 | 0 |
| Radiological pattern (n, %)* |  |  |  |  |
| Ground-glass opacity | 4236 (61.9) | 82 (51.9) | 0.011 | 80 (1.1%) |
| Pleural effusion | 218 (3.2) | 10 (6.3) | 0.030 | 83 (1.2%) |
| Consolidation | 3228 (47.2) | 56 (35.4) | 0.003 | 92 (1.3%) |
| **Treatments (n,%)** |  |  |  |  |
| Glucocorticoid treatment | 2034 (29.7) | 58 (36.7) | 0.057 | 54 (0.8%) |
| LMWH, prophylactic dose, at discharge | 5858 (85.7) | 123 (77.8) | 0.005 | 70 (0.9%) |
| Remdesivir | 31 (0.5) | 0 (0.0) | 0.373 | 89 (1.2%) |
| Lopinavir/Ritonavir | 4533 (66.3) | 87 (55.1) | 0.003 | 32 (0.5%) |
| Interferon | 784 (11.5) | 21 (13.3) | 0.484 | 75 (1.0%) |
| Hydroxychloroquine | 6128 (89.6) | 134 (84.8) | 0.052 | 22 (0.3%) |
| Chloroquine | 256 (3.7) | 4 (2.5) | 0.428 | 57 (0.8%) |
| Tocilizumab | 577 (8.4) | 10 (6.3) | 0.346 | 55 (0.8%) |
| Macrolide | 4291 (62.7) | 75 (47.5) | <0.001 | 45 (0.6%) |
| **Complications (n,%)** |  |  |  |  |
| Bacterial pneumonia | 510 (7.5) | 14 (8.9) | 0.509 | 17 (0.2%) |
| ARDS | 1481 (21.7) | 30 (19.0) | 0.415 | 30 (0.4%) |
| Acute kidney injury | 539 (7.9) | 19 (12.0) | 0.060 | 4 (<0.1%) |
| Acute cardiac injury | 220 (3.2) | 23 (14.6) | <0.001 | 17 (0.2%) |
| Stroke | 21 (0.3) | 1 (0.6) | 0.500 | 16 (0.2%) |
| Sepsis | 136 (2.0) | 1 (0.6) | 0.210 | 4 (<0.1%) |
| Shock | 82 (1.2) | 1 (0.6) | 0.491 | 40 (0.6%) |
| MOF | 37 (0.5) | 2 (1.2) | 0.225 | 21 (0.3%) |

**Appendix . List of the SEMI-COVID-19 Network members Coordinator of the SEMI-COVID-19 Registry:** José Manuel Casas Rojo.

**SEMI-COVID-19 Scientific Committee Members:** José Manuel Casas Rojo, José Manuel Ramos Rincón, Carlos Lumbreras Bermejo, Jesús Millán Núñez-Cortés, Juan Miguel Antón Santos, Ricardo Gómez Huelgas.

**SEMI-COVID-19 Registry Coordinating Center:** S & H Medical Science Service.

**APPENDIX**

Members of the SEMI-COVID-19 Group

H. U. Gregorio Marañón. Madrid

Laura Abarca Casas, Álvaro Alejandre de Oña, Rubén Alonso Beato, Leyre Alonso Gonzalo, Jaime Alonso Muñoz, Crhistian Mario Amodeo Oblitas, Cristina Ausín García, Marta Bacete Cebrián, Jesús Baltasar Corral, Maria Barrientos Guerrero, Alejandro Bendala Estrada, María Calderón Moreno, Paula Carrascosa Fernández, Raquel Carrillo, Sabela Castañeda Pérez, Eva Cervilla Muñoz, Agustín Diego Chacón Moreno, Maria Carmen Cuenca Carvajal, Sergio de Santos, Andrés Enríquez Gómez, Eduardo Fernández Carracedo, María Mercedes Ferreiro-Mazón Jenaro, Francisco Galeano Valle, Alejandra Garcia, Irene Garcia Fernandez-Bravo, María Eugenia García Leoni, Maria Gomez Antunez, Candela González San Narciso, Anthony Alexander Gurjian, Lorena Jiménez Ibáñez, Cristina Lavilla Olleros, Cristina Llamazares Mendo, Sara Luis García, Víctor Mato Jimeno, Clara Millán Nohales, Jesús Millán Núñez-Cortés, Sergio Moragón Ledesma, Antonio Muiño Miguez, Cecilia Muñoz Delgado, Lucía Ordieres Ortega, Susana Pardo Sánchez, Alejandro Parra Virto, María Teresa Pérez Sanz, Blanca Pinilla Llorente, Sandra Piqueras Ruiz, Guillermo Soria Fernández-Llamazares, María Toledano Macías, Neera Toledo Samaniego, Ana Torres do Rego, Maria Victoria Villalba Garcia, Gracia Villarreal, María Zurita Etayo

H. U. 12 de Octubre. Madrid

Paloma Agudo de Blas, Coral Arévalo Cañas, Blanca Ayuso, José Bascuñana Morejón, Samara Campos Escudero, María Carnevali Frías, Santiago Cossio Tejido, Borja de Miguel Campo, Carmen Díaz Pedroche, Raquel Diaz Simon, Ana García Reyne, Lucia Jorge Huerta, Antonio Lalueza Blanco, Jaime Laureiro Gonzalo, Carlos Lumbreras Bermejo, Guillermo Maestro de la Calle, Barbara Otero Perpiña, Diana Paredes Ruiz, Marcos Sánchez Fernández, Javier Tejada Montes

Hospital Universitario Dr. Peset. Valencia

Juan Alberto Aguilera Ayllón, Arturo Artero Mora, María del Mar Carmona Martín, María José Fabiá Valls, Maria de Mar Fernández Garcés, Ana Belén Gómez Belda, Ian López Cruz, Manuel Madrazo López, Elisabet Mateo Sanchis, Jaume Micó Gandia, Laura Piles Roger, Adela Maria Pina Belmonte, Alba Viana García

H. U. La Princesa. Madrid

María Aguilera García, Ester Alonso Monge, Jesús Álvarez Rodríguez, Claudia Alvarez Varela, Miquel Berniz Gòdia, Marta Briega Molina, Marta Bustamante Vega, Jose Curbelo, Alicia de las Heras Moreno, Ignacio Descalzo Godoy, Alexia Constanza Espiño Alvarez, Ignacio Fernández Martín-Caro, Alejandra Franquet López-Mosteiro, Gonzalo Galvez Marquez, María J. García Blanco, Yaiza García del Álamo Hernández, Clara García-Rayo Encina, Noemí Gilabert González, Carolina Guillamo Rodríguez, Nicolás Labrador San Martín, Manuel Molina Báez, Carmen Muñoz Delgado, Pedro Parra Caballero, Javier Pérez Serrano, Laura Rabes Rodríguez, Pablo Rodríguez Cortés, Carlos Rodriguez Franco, Emilia Roy-Vallejo, Monica Rueda Vega, Aresio Sancha Lloret, Beatriz Sánchez Moreno, Marta Sanz Alba, Jorge Serrano Ballester, Alba Somovilla, Carmen Suarez Fernández, Macarena Vargas Tirado, Almudena Villa Marti

H. Miguel Servet. Zaragoza

Gonzalo Acebes Repiso, Uxua Asín Samper, María Aranzazu Caudevilla Martínez, José Miguel García Bruñén, Rosa García Fenoll, Jesús Javier González Igual, Laura Letona Giménez, Mónica Llorente Barrio, Luis Sáez Comet

H. U. Puerta de Hierro. Majadahonda

María Álvarez Bello, Ane Andrés Eisenhofer, Ana Arias Milla, Isolina Baños Pérez, Javier Bilbao Garay, Silvia Blanco Alonso, Jorge Calderón Parra, Alejandro Callejas Díaz, José María Camino Salvador, Mª Cruz Carreño Hernández, Valentín Cuervas-Mons Martínez, Sara de la Fuente Moral, Miguel del Pino Jimenez, Alberto Díaz de Santiago, Itziar Diego Yagüe, Ignacio Donate Velasco, Ana María Duca, Pedro Durán del Campo, Gabriela Escudero López, Esther Expósito Palomo, Ana Fernández Cruz, Esther Fiz Benito, Andrea Fraile López, Amy Galán Gómez, Sonia García Prieto, Claudia García Rodríguez-Maimón, Miguel Ángel García Viejo, Javier Gómez Irusta, Edith Vanessa Gutiérrez Abreu, Isabel Gutiérrez Martín, Ángela Gutiérrez Rojas, Andrea Gutiérrez Villanueva, Jesús Herráiz Jiménez, Pedro Laguna del Estal, Mª Carmen Máinez Sáiz, Cristina Martín Martín, María Martínez Urbistondo, Fernando Martínez Vera, Susana Mellor Pita, Patricia Mills Sánchez, Esther Montero Hernández, Alberto Mora Vargas, Cristina Moreno López, Alfonso Ángel-Moreno Maroto, Victor Moreno-Torres Concha, Ignacio Morrás De La Torre, Elena Múñez Rubio, Ana Muñoz Gómez, Rosa Muñoz de Benito, Alejandro Muñoz Serrano, Jose María Palau Fayós, Ilduara Pintos Pascual, Antonio Ramos Martínez, Isabel Redondo Cánovas del Castillo, Alberto Roldán Montaud, Lucía Romero Imaz, Yolanda Romero Pizarro, Mónica Sánchez Santiuste, David Sánchez Órtiz, Enrique Sánchez Chica, Patricia Serrano de la Fuente, Pablo Tutor de Ureta, Ángela Valencia Alijo, Mercedes Valentín-Pastrana Aguilar, Juan Antonio Vargas Núñez, Jose Manuel Vázquez Comendador, Gema Vázquez Contreras, Carmen Vizoso Gálvez

H. U. de A Coruña. A Coruña

Alicia Alonso Álvarez, Olaya Alonso Juarros, Ariadna Arévalo López, Carmen Casariego Castiñeira, Ana Cerezales Calviño, Marta Contreras Sánchez, Ramón Fernández Varela, Santiago

J. Freire Castro, Ana Padín Trigo, Rafael Prieto Jarel, Fátima Raad Varea, Laura Ramos Alonso, Francisco Javier Sanmartín Pensado, David Vieito Porto

H. Infanta Sofía. S. S. de los Reyes

Rafael del Castillo Cantero, Rebeca Fuerte Martínez, Arturo Muñoz Blanco, José Francisco Pascual Pareja, Isabel Perales Fraile, Isabel Rábago Lorite, Llanos Soler Rangel, Inés Suárez García, Jose Luis Valle López

H. U. Ramón y Cajal. Madrid

Luis Fernando Abrego Vaca, Ana Andréu Arnanz,,Octavio Arce García,,Marta Bajo González, Pablo Borque Sanz, Alberto Cozar Llisto, Sonia de Pedro Baena, Beatriz Del Hoyo Cuenda, María Alejandra Gamboa Osorio, Isabel García Sánchez, Andrés González García, Oscar Alberto López Cisneros, Luis Manzano, Miguel Martínez Lacalzada, Borja Merino Ortiz, Jimena Rey-García, Elisa Riera González, Cristina Sánchez Díaz, Grisell Starita Fajardo, Cecilia Suárez Carantoña, Adrian Viteri Noel

H. U. Infanta Cristina. Parla

Juan Miguel Antón Santos, Ana Belén Barbero Barrera, Coralia Bueno Muiño, Ruth Calderón Hernaiz, Irene Casado Lopez, José Manuel Casas Rojo, Andrés Cortés Troncoso, Mayte De Guzmán García-Monge, Francesco Deodati, Gonzalo García Casasola Sánchez, Elena Garcia Guijarro, Davide Luordo, María Mateos González, Jose A Melero Bermejo, Lorea Roteta García, Elena Sierra Gonzalo, Javier Villanueva Martínez

Hospital Royo Villanova. Zaragoza

Nicolás Alcalá Rivera, Anxela Crestelo Vieitez, Esther del Corral, Jesús Díez Manglano, Isabel Fiteni Mera, Maria del Mar Garcia Andreu, Martin Gerico Aseguinolaza, Claudia Josa Laorden, Raul Martinez Murgui, Marta Teresa Matía Sanz,

Hospital Regional Universitario de Málaga. Málaga

Mª Mar Ayala Gutiérrez, Verónica Andrea Buonaiuto, Rosa Bernal López, José Bueno Fonseca, Luis Francisco Caballero Martínez, Lidia Cobos Palacios, Francis de Windt, Ana Teresa Fernandez-Truchaud Christophel, Paula García Ocaña, Ricardo Gómez Huelgas, Javier Gorospe García, María Dolores López Carmona, Pablo López Quirantes, Almudena López Sampalo, Elizabeth Lorenzo Hernández, Juan José Mancebo Sevilla, Jesica Martin Carmona, Luis Miguel Pérez-Belmonte, Araceli Pineda Cantero, Michele Ricci, Jaime Sanz Cánovas

H. U. La Paz-Cantoblanco-Carlos III. Madrid

Jorge Álvarez Troncoso, Francisco Arnalich Fernández, Francisco Blanco Quintana, Carmen Busca Arenzana, Sergio Carrasco Molina, Aranzazu Castellano Candalija, Germán Daroca

Bengoa, Alejandro de Gea Grela, Alicia de Lorenzo Hernández, Carmen Fernández Capitán, Maria Francisca García Iglesias, Carmen Rosario Herrero Gil, Juan María Herrero Martínez, Víctor Hontañón, Maria Jesús Jaras Hernández, Carlos Lahoz, Cristina Marcelo Calvo, Juan Carlos Martín Gutiérrez, Monica Martinez Prieto, Elena Martínez Robles, Araceli Menéndez, Araceli Menéndez Saldaña, Alberto Moreno Fernández, Jose Maria Mostaza Prieto, Ana Noblejas Mozo, Esmeralda Palmier Peláez, Marina Palomar Pampyn, Maria Angustias Quesada Simón, Juan Carlos Ramos Ramos, Luis Ramos Ruperto, Aquilino Sánchez Purificación, Teresa Sancho Bueso, Raquel Sorriguieta Torre, Clara Itziar Soto Abanedes, Yeray Untoria Tabares, Marta Varas Mayoral, Julia Vásquez Manau

Consorcio H. G. U. de Valencia. Valencia

Francesc Puchades, Juan José Tamarit García

H. de Cabueñes. Gijón

Ana María Álvarez Suárez, Carlos Delgado Vergés, Rosa Fernandez-Madera Martínez, Eva Fonseca Aizpuru, Alejandro Gómez Carrasco, Cristina Helguera Amezua, Juan Francisco López Caleya, María del Mar Martínez López, Aleida Martínez Zapico, Carmen Olabuenaga Iscar, María Luisa Taboada Martínez, Lara María Tamargo Chamorro

H. de Sagunto. Sagunto

Zineb Karroud Zamrani, Jose Maria Pascual Izuel, Enrique Rodilla

H. U. San Agustin. Avilés

Andrea Álvarez García, Víctor Arenas García, Alba Barragán Mateos, Demelsa Blanco Suárez, María Caño Rubia, Jaime Casal Álvarez, David Castrodá Copa, José Ferreiro Celeiro, Natalia García Arenas, Raquel García Noriega, Joaquin Llorente García, Irene Maderuelo Riesco, Paula Martinez Garcia, Maria Jose Menendez Calderon, Diego Eduardo Olivo Aguilar, Marta Nataya Solís Marquínez, Luis Trapiella Martínez, Andrés Astur Treceño García, Juan Valdés Bécares

H. U. San Juan de Alicante. San Juan de Alicante

David Balaz, David Bonet Tur, Carles García Cervera, David Francisco García Núñez, Vicente Giner Galvañ, Angie Gómez Uranga, Javier Guzmán Martínez, Isidro Hernández Isasi, Lourdes Lajara Villar, Juan Manuel Núñez Cruz, Sergio Palacios Fernández, Juan Jirge Peris García, Andrea Riaño Pérez, José Miguel Seguí Ripoll, Philip Wikman-Jorgensen

H. del Henares. Coslada

Jesús Ballano Rodríguez-Solís, Luis Cabeza Osorio, María del Pilar Fidalgo Montero, Mª Isabel Fuentes Soriano, Erika Esperanza Lozano Rincon, Ana Martín Hermida, Jesus Martinez Carrilero, Jose Angel Pestaña Santiago, Manuel Sánchez Robledo, Patricia Sanz Rojas, Nahum Jacobo Torres Yebes, Vanessa Vento,

H. San Pedro. Logroño

Diana Alegre González, Irene Ariño Pérez de Zabalza, Sergio Arnedo Hernández, Jorge Collado Sáenz, Beatriz Dendariena, Marta Gómez del Mazo, Iratxe Martínez de Narvajas Urra, Sara Martínez Hernández, Estela Menendez Fernández, Jose Luís Peña Somovilla, Elisa Rabadán Pejenaute

Hospital Universitari de Bellvitge. L'Hospitalet de Llobregat

Francesc Formiga, Abelardo Montero, Manuel Rubio-Rivas, Sergi Yun Viladomat

H. Juan Ramón Jiménez. Huelva

Francisco Javier Bejarano Luque, Francisco Javier Carrasco-Sánchez, Mercedes de Sousa Baena, Jaime Díaz Leal, Aurora Espinar Rubio, Maria Franco Huertas, Juan Antonio García Bravo, Andrés Gonzalez Macías, Encarnación Gutiérrez Jiménez, Constantino Lozano Quintero, Carmen Mancilla Reguera, Francisco Javier Martínez Marcos, Francisco Muñoz Beamud, Maria Perez Aguilera, Alícia Perez Jiménez, Virginia Rodríguez Castaño, Alvaro Sánchez de Alcazar del Río, Leire Toscano Ruiz

H. U. Reina Sofía. Córdoba

Antonio Pablo Arenas de Larriva, Pilar Calero Espinal, Javier Delgado Lista, María Jesús Gómez Vázquez, Jose Jiménez Torres, Laura Martín Piedra, Javier Pascual Vinagre, María Elena Revelles Vílchez, Juan Luis Romero Cabrera, José David Torres Peña

H. G. U. de Alicante. Alicante

Mar García Navarro, Ana Martí Pastor, José Manuel Ramos Rincón, Diego Torrús Tendero

H. Santa Marina. Bilbao

Maria Areses Manrique, Ainara Coduras Erdozain, Ane Labirua-Iturburu Ruiz

Hospital de Urduliz Alfredo Espinosa. Urdúliz

María Aparicio López, Asier Aranguren Arostegui, Paula Arriola Martínez, Gorka Arroita Gonzalez, Mª Soledad Azcona Losada, Miriam García Gómez, Eduardo Garcia Lopez, Amalur Iza Jiménez, Alazne Lartategi Iraurgi, Esther Martinez Becerro, Itziar Oriñuela González, Isabel María Portales Fernández, Pablo Ramirez Sánchez, Beatriz Ruiz Estévez, Cristian Vidal Núñez

C. H. U. de Albacete. Albacete

Jose Luis Beato Pérez, Maria Lourdes Sáez Méndez

Hospital Universitario Ntra Sra Candelaria. Santa Cruz de Tenerife

Lucy Abella, Andrea Afonso Díaz, Selena Gala Aguilera Garcia, Marta Bethencourt Feria, Eduardo Mauricio Calderón Ledezma, Sara Castaño Perez, Guillermo Castro Gainett, José Manuel del Arco Delgado, Joaquín Delgado Casamayor, Diego Garcia Silvera, Alba Gómez Hidalgo, Carolina Hernández Carballo, Rubén Hernández Luis, Francisco Javier Herrera Herrera, Maria del mar Lopez Gamez, Julia Marfil Daza, María José Monedero Prieto, María Blanca Monereo

Muñoz, María de la Luz Padilla Salazar, Daniel Rodríguez Díaz, Alicia Tejera, Laura Torres Hernández

C. Asistencial de Zamora. Zamora

Carlos Aldasoro Frias, Luis Arribas Perez, María Esther Fraile Villarejo, Beatriz Garcia Lopez, Victor Madrid Romero, Victoria Palomar Calvo, Sara Pintos Otero, Carlota Tuñón de Almeida

H. San Juan de la Cruz. Úbeda

Marcos Guzmán Garcia, Francisco Javier Vicente Hernández

Hospital Costa del Sol. Marbella

Victoria Augustín Bandera

H. U. Virgen de las Nieves. Granada

Pablo Conde Baena, Joaquin Escobar Sevilla, Laura Gallo Padilla, Patricia Gómez Ronquillo, Pablo González Bustos, María Navío Botías, Jessica Ramírez Taboada, Mar Rivero Rodrígez

Hospital Clínico de Santiago. Santiago de Compostela

Maria del Carmen Beceiro Abad, Maria Aurora Freire Romero, Sonia Molinos Castro, Emilio Manuel Paez Guillan, María Pazo Nuñez, Paula Maria Pesqueira Fontan,

Hospital HLA Moncloa. Madrid

Guillermo Estrada, Teresa Garcia Delange, Isabel Jimenez Martinez, Carmen Martinez Cilleros, Nuria Parra Arribas,

Hospital Marina Baixa. Villajoyosa

Javier Ena, Jose Enrique Gómez Segado

Complejo Asistencial Universitario de León. León

Rosario Maria García die, Manuel Martin Regidor, Angel Luis Martínez Gonzalez, Alberto Muela Molinero, Raquel Rodríguez Díez, Beatriz Vicente Montes

H. U. Marqués de Valdecilla. Santander

Marta Fernández-Ayala Novo, José Javier Napal Lecumberri, Nuria Puente Ruiz, Jose Riancho, Isabel Sampedro Garcia

H. U. C. de Asturias. Oviedo

Victor Asensi Alvarez, Itxasne Cabezón Estévanez, Maria Folgueras Gómez, Lucía Meijide Rodríguez, Claudia Moran Castaño, Noelia Morán Suárez, Sara Rodríguez Suárez, Silvia Suárez Díaz, Lucia Suárez Pérez, Carlos Vázquez, Carmen Yllera Gutiérrez

H. U. Virgen del Rocío. Sevilla

Verónica Alfaro Lara, Bosco Baron Franco, Máximo Bernabeu-Wittel, Concepción Conde Guzmán, Juan Delgado de la Cuesta, Pablo Díaz Jiménez, Fátima Espinosa Torre, Rosa María Gámez Mancera, Luis Giménez Miranda, Aurora González Estrada, Sonia Gutiérrez Rivero, Carlos Hernandez Quiles, Carlos Jiménez de Juan, Julia Lanseros Tenllado, María del Carmen López Ríos, María Nieto, Santiago Rodríguez Suárez, Jara Eloisa Ternero Vega

Hospital Clinic Barcelona. Barcelona

Júlia Calvo Jiménez, Aina Capdevila Reniu, Irene Carbonell De Boulle, Emmanuel Coloma Bazán, Joaquim Fernández Solà, Cristina Gabara Xancó, Joan Ribot Grabalosa, Olga Rodríguez Núñez

C. H. U. de Ferrol. Ferrol

Hortensia Alvarez Diaz, Tamara Dalama Lopez, Estefania Martul Pego, Carmen Mella Pérez, Ana Pazos Ferro, Sabela Sánchez Trigo, Dolores Suarez Sambade, Maria Trigas Ferrin, Maria del Carmen Vázquez Friol, Laura Vilariño Maneiro,

H. U. La Fe. Valencia

Ricardo Gil Sánchez, Marta Jiménez Escrig, Laura Parra Gómez, José Antonio Todolí Parra

H. Francesc de Borja. Gandia

Leyre Jorquer Vidal, M Angeles Martinez Pascual

H. U. del Vinalopó. Elche

Francisco Amorós Martínez, Erika Ascuña Vásquez, Jose Carlos Escribano Stablé, Adriana Hernández Belmonte, Ana Maestre Peiró, Raquel Martínez Goñi, M.Carmen Pacheco Castellanos, Bernardino Soldan Belda, David Vicente Navarro

H. U. Son Llàtzer. Palma de Mallorca

Andrés de la Peña Fernández

Hospital Valle del Nalón. Riaño (Langreo)

Sara Fuente Cosío, César Manuel Gallo Álvaro, Julia Lobo García, Antía Pérez Piñeiro,

H. U. Torrevieja. Torrevieja

Julio César Blázquez Encinar

Hospital del Tajo. Aranjuez

Ruth Gonzalez Ferrer, Raquel Monsalvo Arroyo

H. de Mataró. Mataró

Raquel Aranega González, Ramon Boixeda, Carlos Lopera Mármol, Marta Parra Navarro, Ainhoa Rex Guzmán, Aleix Serrallonga Fustier

H. de Poniente. Almería

Juan Antonio Montes Romero, Encarna Sánchez Martín, Jose Luis Serrano Carrillo de Albornoz, Manuel Jesus Soriano Pérez

H. Sierrallana. Torrelavega

Cristina Amado Fernández, Tomás de Vega Santos, Cristina Limia, Lucia Paz Fajardo, Andrea Tejero Fernández, Reina Valle Bernad

H. G. U. de Castellón. Castellón de la Plana

Jorge Andrés Soler, Marián Bennasar Remolar, Alejandro Cardenal Álvarez, Daniela Díaz Carlotti, María José Esteve Gimeno, Sergio Fabra Juana, Paula García López, María Teresa Guinot Soler, Daniela Palomo de la Sota, Guillem Pascual Castellanos, Ignacio Pérez Catalán, Celia Roig Martí, Paula Rubert Monzó, Javier Ruiz Padilla, Nuria Tornador Gaya, Jorge Usó Blasco

H. Moisès Broggi. Sant Joan Despí

Jose Loureiro Amigo, Melani Pestaña Fernández

Hospital Platón. Barcelona

Ana Suarez Lombraña

Hospital Doctor José Molina Orosa. Arrecife (Lanzarote)

Virginia Herrero García, Berta Román Bernal

H. U. Lucus Augusti. Lugo

Raquel Gómez Méndez, Ana Rodríguez Álvarez

H. G. U. de Elda. Elda

Carmen Cortés Saavedra, Jennifer Fernández Gómez, Borja González López, María Soledad Hernández Garrido, Ana Isabel López Amorós, Maria de los Reyes Pascual Pérez, Andrea Torregrosa García

H. de la Axarquía. Vélez- Málaga

Antonio Lopez Ruiz

H. Nuestra Señora del Prado. Talavera de la Reina

Sonia Casallo Blanco, Jeffrey Oskar Magallanes Gamboa

H. Santa Bárbara. Soria

Marta Leon Tellez

H. Nuestra Señora de Sonsoles. Ávila

Alaaeldeen Abdelhady Kishta

H. U. Severo Ochoa. Leganés

Yolanda Casillas Viera, Lucía Cayuela Rodríguez, Gema Flox Benitez, Laura García Escudero, Juan Martin Torres, Patricia Moreira Escriche, Susana Plaza Canteli, M Carmen Romero Pérez

H. San Pedro de Alcántara. Cáceres

Angela Agea Garcia, Javier Galán González, Luis Gámez Salazar, Eva Garcia Sardon, Antonio González Nieto, Itziar Montero Días, Selene Núñez Gaspar, Alvaro Santaella Gomez

H. U. de Canarias. Santa Cruz de Tenerife

Julio Cesar Alvisa Negrin, José Fernando Armas González, Lourdes González Navarrete, Iballa Jiménez, María Candelaria Martín González, Miguel Nicolas Navarrete Lorite, Paula Ortega Toledo, Onán Pérez Hernández, Alina Pérez Ramírez,

H. Asepeyo Coslada. Coslada

Alejo Erice Calvo-Sotelo

Hospital de Palamós. Palamós

Anabel Martin-Urda Diez-Canseco

H. de Pozoblanco. Pozoblanco

José Nicolás Alcalá Pedrajas, Antonia Márquez García, Inés Vargas

H. U. del Sureste. Arganda del Rey

Ana Belén Mancebo Plaza, Arturo Noguerado Asensio, Bethania Pérez Alves, Jon Cabrejas Ugartondo, Natalia Vicente López

H. Parc Tauli. Sabadell

Francisco Epelde, Isabel Torrente

H. U. Puerta del Mar. Cádiz

Susana Fabiola Pascual Perez

Hospital do Salnes. Vilagarcía de Arousa

Cristina Pérez García

Hospital Insular de Gran Canaria. Las Palmas G. C. Carlos Jorge Ripper

Hospital Quironsalud A Coruña. A Coruña

Hector Meijide Miguez

Complejo Asistencial de Segovia. Segovia

Daniel Monge Monge

C. H. U. de Badajoz. Badajoz

Gema María García García

C. A. U. de Salamanca. Salamanca

José Ángel Martín Oterino

Hospital Torrecárdenas. Almería

Luis Felipe Díez García

H. U. Rafael Méndez. Lorca

Ana Isabel Peláez Ballesta

H. U. Infanta Leonor. Madrid

Beatriz Mestre Gómez

Hospital Público de Monforte de Lemos. Monforte de Lemos Manuel Lorenzo López Reboiro

H. Virgen del Mar. Madrid

Maria jesus Gonzalez Juarez

Hospital Perpetuo Socorro. Badajoz

Maria Jose Luque Calderon

H. de Éibar. Éibar

Esperanza Montero Aparicio

Consorci Sanitari de Terrassa. Terrassa

Anna Fajardo Modol

H. La Fuenfría. Cercedilla

Isabel Rodriguez Fraile

H. Comarcal de Blanes. Blanes

Pere Comas Casanova

Santa Ana. Motril

Jesús Palomares Rodríguez

H. de Zafra. Zafra

Juana Carretero Gómez

H. G. U. Los Arcos del Mar Menor. San Javier Diana Piñar Cabezos

H. Clinico San Carlos. Madrid

Manuel Mendez Bailon

C. H. U. de Cáceres. Cáceres

Marta Correa Matos

Fundació Sant Hospital de la Seu d'Urgell. La Seu d'Urgell Luis Enrique Cajamarca Calva

H. U. Santa Lucía. Cartagena

Pedro José García López

H. García Orcoyen. Estella

M. Carmen Martínez Velasco

Hospital Comarcal de Inca. Inca

María Soledad Sanz Parras

H. General U. Reina Sofia. Murcia

José Joaquín Hernández Roca

Hospital de Barbastro. Barbastro

Juan Salas Jarque

Hospital del Vendrell. El Vendrell

Ana Lacal Martinez

H. U. Río Hortega. Valladolid

Luis Corral Gudino

Centro Médico de Asturias. Oviedo

Fidel Asensio Fierro

H. San Juan de Dios del Aljarafe. Bormujos Maria de la Luz Calero Bernal

H. U. de Gran Canaria Dr. Negrin. Las Palmas G. C. Alicia Conde

Hospìtal de Montilla. Montilla

Adrián Montaño Martínez

Hospital Infanta Margarita. Cabra

María Esther Guisado Espartero

H. Virgen de los Lirios. Alcoy (Alicante)

Mª José Esteban Giner

H. U. Rey Juan Carlos. Móstoles

José Antonio Rueda Camino

Clinica San Miguel. Pamplona. Pamplona Raquel Rodil

IAS Sta. Caterina. Salt

Sara Garcia Torras

H. Insular Ntra. Sra. de los Reyes. Valverde (El Hierro) Ana María Torres Vega

Hospital Alto Guadalquivir. Andújar

Begoña Cortés Rodríguez

H. U. Virgen de la Victoria. Málaga

María José Benítez Toledo

H.U. de la Plana. Vila-Real (Castellón) Iris Pedrola Gorrea
